# Endovascular treatment of a linguofacial trunk pseudoaneurysm after tonsillectomy

**DOI:** 10.5935/1808-8694.20130094

**Published:** 2015-10-08

**Authors:** Luciano Manzato, Felipe Padovani Trivelato, Alexandre Yugo Holayama Alvarenga, Marco Túlio Rezende, Alexandre Cordeiro Ulhôa

**Affiliations:** a MD (Fellow - Interventional neuroradiology - Hospital das Clínicas - Federal University of Minas Gerais).; b MD (Interventional neuroradiologist - Hospital das Clínicas - Federal University of Minas Gerais).; c MD (Resident - Neurosurgery - Hospital das Clínicas - Federal University of Minas Gerais). Division of Interventional Neuroradiology - Hospital das Clínicas - Federal University of Minas Gerais - Belo Horizonte - Minas Gerais - Brazil.

**Keywords:** aneurysm, false, embolization, therapeutic, endovascular procedures, tonsillectomy

## INTRODUCTION

Tonsillectomy is one of the most often performed procedures in ear-nose-throat departments. Many different complications may arise after this procedure, including infection, prolonged pain, cranial nerves lesions causing voice alterations or difficulty to swallow. The most common serious one is postoperative hemorrhage involving around 3% of the cases^1^. The most severe hemorrhages are due to arterial dissections or pseudoaneurysms^2^.

Pseudoaneurysms may arise after localized arterial laceration caused by blunt or penetrating trauma, including traction and thermal damage produced by electrocautery. Post-tonsillectomy hemorrhages (PTH) from pseudoaneurysms located at the lingual, facial and internal carotid arteries have been described^2^, ^3^, ^4^, ^5^. The treatment options include local maneuvers, surgical ligation and endovascular embolization. We report a case of a 19-year-old female with recurrent severe bleeding caused by a pseudoaneurysm of the left linguofacial trunk.

## CASE REPORT

A 19-year-old female underwent a left sided tonsillectomy because of recurrent tonsillitis. There were no perioperative complications and she made an uneventful recovery. After 20 days she presented with oral bleeding arising from the operative site that was managed with suture ligatures and packing with gauze. The day after she experienced other 2 episodes of extensive bleeding.

She was readmitted to hospital hemodynamically unstable, where she was intubated. An emergent angiogram under general anesthesia was performed to exclude vascular injury. The examination showed no internal carotid artery (ICA) injury, but the selective injection from the external carotid artery (ECA) revealed a pseudoaneurysm arising from the left linguofacial trunk.

After the diagnostic studies of the craniocervical vasculature, the left external carotid artery was catheterized with a 6F Guider catheter (Stryker Co, MI, USA). Coaxially an Excelsior SL10 microcatheter (Stryker Co, MI, USA) was advanced over a 0.014-inch Transend wire (Stryker Co, MI, USA) to a position just beyond the pseudoaneurysm.

**Figure 1:**
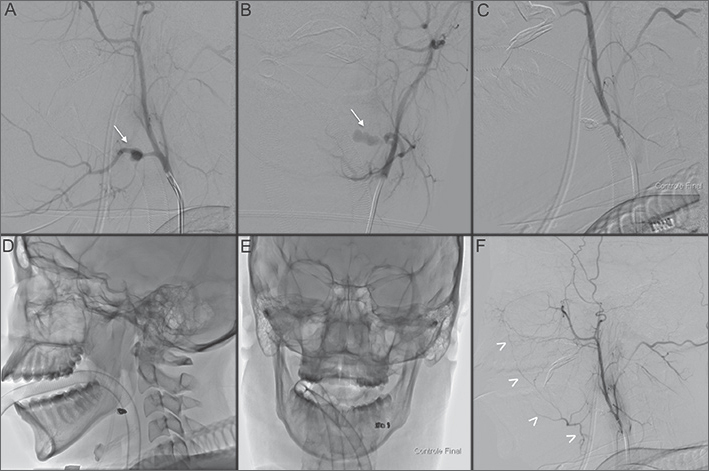
A and B: Left external carotid angiography (lateral and AP projections) showing a pseudoaneuryms of the linguofacial trunk (arrows); C: Final control after internal trapping; D and E: Cast of coils; F: Retrograde flling of linguofacial vascular territory via internal maxillary artery anastomosis (arrowheads).

It was decided to occlude both the parent vessel (linguofacial trunk) and the pseudoaneurysm. Embolization was performed with seven Guglielme detachable coils. Final control angiogram showed total exclusion of the aneurysm and retrograde filling of linguofacial vascular territory via internal maxillary artery anastomosis (Figure 1).

There were no complications related to the endovascular procedure. She was discharged after 5 days with no more episodes of bleeding.

## DISCUSSION

Postoperative hemorrhage is the most common serious complication of tonsillectomy and its incidence is around 3%. They represent the major cause of prolonged post procedural hospitalization^1^. Serious post-tonsillectomy hemorrhage seems to be more common in children up to 8 years of age and in cases of bleeding after 24 hours of the procedure. Although rare, pseudoaneurysms of the carotid artery and its branches following tonsillectomy are clinically significant lesions^2^, ^3^, ^4^, ^5^.

Pseudoaneurysms may arise after localized arterial laceration caused by blunt or penetrating trauma. While these lesions lack the three layers of the arterial wall, they are also called false aneurysms. Luminal blood leaks out through the damaged arterial wall into the surrounding soft tissues and forms a pseudoaneurysm sac that communicates directly to the arterial lumen^2^, ^3^, ^5^.

Arterial injury can be due to anatomic variability of the great arteries and their close relation to the tonsil, and in part to the generous arterial supply to the palatine tonsils, which includes the descending palatine artery arising from the internal maxillary artery, the ascending pharyngeal artery, the dorsal lingual artery arising from the lingual artery, and the ascending palatine artery and tonsillar artery originating from the facial artery (main supply)^1^, ^6^.

Some anatomical features existent at pediatric population as smaller anatomy and thinner pharyngeal muscles determines a higher risk of arterial trauma during tonsillectomy. Pseudoaneurysms after tonsillectomy are very rare in adults^3^.

A literature search yielded 23 additional cases of traumatic pseudoaneurysms after tonsillectomy. The lesions were located at the lingual artery in 10 cases (43.5%), internal carotid artery in three cases (13%), facial artery in two cases (8.7%), external carotid artery trunk in two cases, linguofacial trunk (which is present in up to 20% of cases) in two cases and not specified in three cases^2^, ^3^, ^4^, ^5^.

In 20 of the reported cases, treatment consisted in endovascular approach in 13, surgical in 6, and combined in one case, a patient with an internal carotid artery pseudoaneurysm that underwent surgical resection after distal endovascular occlusion. Among endovascular treated patients, 11 patients underwent internal trapping with coils, one underwent stenting of the ICA and one underwent selective coiling of a linguofacial trunk pseudoaneurysm that resulted in rebleeding and surgical reintervention^2^, ^3^, ^4^, ^5^.

Treatment options for post-tonsillectomy hemorrhage include local maneuvers, surgical ligation and endovascular embolization. The first one is associated with high failure rates and repetitive bleeding, thus not recommended. Direct cervical surgical access carries the risk of injury to superior laryngeal or vagus nerves, stroke and diminished vascular reserve in the arterial distribution of the vessels ligated. Moreover, serious bleeding may not stop after proximal ligation of the external carotid artery or its branches^5^.

Endovascular treatment of PTH has three main advantages: first, the diagnostic evaluation can be combined with direct therapeutic intervention; second, embolization is more selective; and third, this method is less mutilating and has less risk of damaging the vagal and accessory nerves^1^, ^3^, ^5^.

While leading with saccular, true aneurysms, selective treatment is always desired, once it preserves the parent artery. On the other hand, the rich collateral blood supply in this area makes internal trapping the best approach, because the main risk of selective occlusion is that the pseudoaneurysm wall may not provide a permanent barrier to the movement of the embolic agent. Moreover, the durability of this treatment is uncertain and recurrent hemorrhage is possible^5^.

Unilateral proximal occlusion of facial and lingual arteries is generally clinically well tolerated. In the case of lingual artery, there are anastomoses with the contralateral lingual and ipsilateral facial via the submental branch. In the case of facial artery, there are anastomoses with superior thyroid via its infrahyoid branch, and also side-to-side anastomoses with its contralateral partner. Other important connections include the internal maxillary, transverse facial, and distal ophthalmic branches^6^.

Internal trapping with coils of the involved segment is the treatment of choice, because inadvertent distal migration of embolic agents, such as glue or PVA particles, is not desired and is associated with additional complications. For example, the branches of the tip of the tongue are effectively end arteries, and distal occlusion can produce ischemic necrosis of the tip of the tongue^6^.

When facing post-tonsillectomy hemorrhage, emergent angiography to rule out vascular lesions is strongly recommended. Once a pseudoaneurysm is identified, internal trapping shows to be an effective, definitive and safe treatment^3^, ^5^.

## FINAL REMARKS

Pseudoaneurysms are life-threatening, and besides rare, they should be considered in severe post tonsillectomy hemorrhage. The most common artery involved is the lingual, and in a very few cases the facial or linguo-facial trunk. In these cases of hemorrhage, arteriography is strongly recommended because it can be diagnostic and therapeutic at the same procedure. Endovascular treatment is safe, effective and definite.

## References

[1] Opatowsky MJ, Browne JD, McGuirt WF, Morris PP (2001). Endovascular treatment of hemorrhage after tonsillectomy in children. AJNR Am J Neuroradiol.

[2] Atmaca S, Belet U, Baris S (2012). Post-tonsillectomy pseudoaneurysm of the linguofacial trunk: An ENT surgeon's nightmare. Int J Pediatr Otorhinolaryngol Extra.

[3] Van Cruijsen N, Gravendeel J, Dikkers FG (2008). Severe delayed posttonsillectomy haemorrhage due to a pseudoaneurysm of the lingual artery. Eur Arch Otorhinolaryngol.

[4] Karas DE, Sawin RS, Sie KC (1997). Pseudoaneurysm of the external carotid artery after tonsillectomy. A rare complication. Arch Otolaryngol Head Neck Surg.

[5] Windfuhr JP, Sesterhenn AM, Schloendorff G, Kremer B (2010). Post-tonsillectomy pseudoaneurysm: an underestimated entity?. J Laryngol Otol.

[6] Lasjaunias P, Berestein A, ter Brugge KG (2001). Surgical Neuroangiography, Volume 1: Clinical vascular anatomy and variations.

